# Test-retest reproducibility of a food frequency questionnaire (FFQ) and estimated effects on disease risk in the Norwegian Women and Cancer Study (NOWAC)

**DOI:** 10.1186/1475-2891-5-4

**Published:** 2006-01-31

**Authors:** Christine L Parr, Marit B Veierød, Petter Laake, Eiliv Lund, Anette Hjartåker

**Affiliations:** 1 Institute of Basic Medical Sciences, Department of Biostatistics, University of Oslo, P.O. Box 1122 Blindern, N-0317 Oslo, Norway; 2 Institute of Community Medicine, University of Tromsø, N-9037 Tromsø, Norway

## Abstract

**Background:**

The Norwegian Women and Cancer Study (NOWAC) is a national population-based cohort study with 102 443 women enrolled at age 30–70 y from 1991 to 1997. The present study was a methodological sub-study to assess the test-retest reproducibility of the NOWAC food frequency questionnaire (FFQ), and to study how measurement errors in the data can affect estimates of disease risk.

**Methods:**

A random sample of 2000 women aged 46–75 y was drawn from the cohort in 2002. A self-instructive health and lifestyle questionnaire with a FFQ section was mailed to the same subjects twice (test-retest), about three months apart, with a response rate of 75%. The FFQ was designed to assess habitual diet over the past year. We assess the reproducibility of single questions, food groups, energy, and nutrients with several statistical measures. We also demonstrate the method of regression calibration to correct disease risk estimates for measurement error. Alcohol intake (g/day) and high blood pressure (yes/no) is used in the example.

**Results:**

For single foods there were some indications of seasonal reporting bias. For food groups and nutrients the reliability coefficients ranged from 0.5–0.8, and Pearson's *r*, Spearman's *r*_*s*_, and two intraclass correlation coefficients gave similar results. Although alcohol intake had relatively high reproducibility (*r *= 0.72), odds ratio estimates for the association with blood pressure were attenuated towards the null value compared to estimates corrected by regression calibration.

**Conclusion:**

The level of reproducibility observed for the FFQ used in the NOWAC study is within the range reported for similar instruments, but may attenuate estimates of disease risk.

## Background

In epidemiological studies of diet and disease, food frequency questionnaires (FFQs) have long been the dominant method for measuring dietary intake. Researchers now recognize that data from FFQs and other dietary assessment methods can have substantial measurement errors, both systematic and random, which may lead to biased disease risk estimates [1]. This has become a central point in the discussion of conflicting research results on diet-cancer associations [2,3]. It has been argued that the potential for progress may be greater in understanding the nature of errors and developing statistical correction methods, rather than new collection methods for dietary data [4]. Numerous studies have been published from the nutrition community on the reproducibility and validity of different dietary assessment instruments. A selection of validation studies is found in the Dietary Validation/Calibration register maintained by the National Cancer Institute [5]. However, few studies estimate effects of reproducibility and validity on outcomes. This could be due to little collaboration with epidemiological and statistical communities, or the fact that analytical tools for handling error in dietary data are still in an early stage of development and not easily accessible.

As part of a larger validation study, the main aim of the present study is to assess the test-retest reproducibility of the FFQ developed for the prospective Norwegian Women and Cancer study (NOWAC). Reproducibility, or how consistently FFQ measurements can be repeated on the same subjects, is a useful first estimate of questionnaire performance [6]. The reproducibility is analysed for single questions, food groups, energy, and nutrients. We use several statistical measures to capture different aspects of reproducibility and to facilitate the comparison with other studies. We also examine potential effects on outcomes by comparing disease risk estimates based on exposure data from the test and retest, and disease risk estimates corrected for measurement error by regression calibration.

## Methods

### Study design

NOWAC is a national population-based cohort study with 102 443 women enrolled at age 30–70 years from 1991 to 1997. The cohort has been described in detail elsewhere [7]. Updated information can be found on the NOWAC web-site [8]. NOWAC includes the Norwegian sub-cohort in the European Prospective Investigation into Cancer and Nutrition (EPIC). The present methodological sub-study was undertaken to assess the reproducibility of the food frequency questionnaire developed for NOWAC and the Norwegian part of the EPIC study. The FFQ covers four consecutive pages within a larger self-instructive health and lifestyle questionnaire (eight pages) that is administered by post and optically read. The same questionnaire was mailed twice (test and retest) to the same subjects, about three months apart in February/March and May/June 2002. A letter of invitation and a return envelope with pre-paid postage were included. Non-responders received up to two written reminders for each questionnaire. No rewards were given to participants.

### Subjects

In 2002 a follow-up questionnaire was mailed to 36 000 women from the cohort aged 46–75 years. Those who returned the questionnaire within four weeks (n = 14 817) were taken as the sampling frame, from which a random sample of 2000 women was drawn for the reproducibility study. The sampling was done by Statistics Norway using the national population registry, which identifies all Norwegian residents by a unique 11-digit national person number incorporating birth date and sex. Information about name, address, emigration and death is continuously updated based on mandatory registration and notification to the registry. To retain confidentiality the person number was replaced by a serial number on the letter of invitation and questionnaire, and in the data files. The study was approved by the Regional Committee for Medical Research Ethics, Northern Norway, and the license for data storage and processing was issued by the national Data Inspectorate.

In the random sample of 2000 women, five had not given an informed consent to further contact and were excluded. The retest questionnaire was returned by 1496 (75%) of the 1995 women. One test questionnaire was not available at the time of analysis, and seven women with null energy intake in either test or retest were excluded. Thus, 1488 respondents with two FFQ measurements could be included in the reproducibility analyses. Background characteristics were compared for the respondents and 1994 women from the original sample to check for selection bias. Except for age, all characteristics were based on self-reported information in the test questionnaire.

The reproducibility analysis of single questions in the FFQ included pairs of test-retest responses without missing values, so the number of subjects included varied. The analysis of food groups and nutrients included 1370 women (92%) who answered at least 50% of the frequency questions and had energy intake in the range 2500–15000 kJ in both test and retest. Similar inclusion criteria have previously been used in NOWAC [9]. The effects of exposure measurement error on disease risk estimates were investigated using the 1370 subjects from the food group and nutrient analysis, who also had completed a question about high blood pressure. Those who answered "yes" or "no" to this question in both test and retest, were defined as cases (n = 301) and controls (n = 712), respectively. Subjects with inconsistent or missing answers were excluded.

### The food frequency questionnaire (FFQ)

The FFQ was designed to assess habitual diet over the past year, with emphasis on fish consumption and a traditional diet in the study population. Questions were asked about the intake of milk, coffee, orange juice, soft drinks, yoghurt, breakfast cereal, bread, fat on bread, toppings for open sandwiches (jam, cheeses, meat and fish products), fruit, vegetables, potatoes, rice, pasta, rice porridge, fish and fish products, shellfish, condiments and sauces for fish, meat and poultry, eggs, ice cream, cakes, desserts, chocolate, snacks, alcoholic beverages, and dietary supplements. Similar items were grouped together in blocks with question headings. The response options were predefined and listed in increasing order with check-boxes to facilitate completion and optical reading. For example, the items listed under the question "How often do you eat fruit?" were "apples/pears", "oranges", "bananas", and "other fruit" with the following options: "never/rarely", "1–3 per month", "1 per week", "2–4 per week", "5–6 per week", "1 per day", and "2+ per day". The first alternative for consumption frequencies was always "never/rarely", but the number of options ranged from 4 to 7 depending on the food. When convenient, the questions were phrased in terms of natural units, such as glasses (milk, fruit juice, soft drinks, and wine), cups (coffee), slices (bread), or number (eggs and potatoes). Separate questions about the usual amounts consumed were included for fat on bread, vegetables, fish and fish products, sauces and condiments for fish, meat and meat products, ice cream, chocolate, and cod liver oil supplements. The number of response options ranged from 3 to 5 with units in pieces, slices, decilitres, florets (broccoli and cauliflower), or spoonfuls. The dietary intake computations included a total of 132 questions in the FFQ (consumption frequencies = 91, types of fat used on bread = 7, amounts = 28, and time of year for the consumption of different species of fish = 6). A detailed list of the food items, including a specification of those with a separate amount question, can be found in Additional file 1. The original version of the test-retest FFQ is shown in Additional file 2.

### Computation of dietary intake

The daily intake of food groups, energy, and nutrients was computed using an analysis program developed at the Institute of Community Medicine, University of Tromsø, for SAS software. The program was run with an updated file version of the food composition table for Norway [10]. Broader categories of foods (e.g. "apples/pears") were split into single foods according to frequency weights (e.g. 80% apples and 20% pears) derived from 24-hour dietary recalls in a random sample of women within NOWAC [11,12]. For season specific frequencies (ice cream, fish, and cod liver oil supplements) the average for the whole year was used. Missing frequencies were treated as null intake, and missing portion sizes were substituted by the smallest portion for a conservative intake estimate. Standard portion sizes and standard weights were taken from official tables for Norway [13]. The type of fat used on bread was taken into account in the calculations, but not fat in cooking since the intake of fried and cooked foods was computed using values for prepared foods in the food composition table. The only dietary supplement included was cod liver oil (liquid and capsules), which is commonly used in Norway as a source of vitamin A, vitamin D, and long-chain ω-3 fatty acids. The food groups were based on the classification system in the EPIC-SOFT program for conducting 24-hour dietary recalls in the EPIC study [14], but with some modifications. Peanuts and potato chips were added to the EPIC group "Sugar and confectionary" and called "Sweets and salty snacks". The EPIC groups "Potatoes and other tubers" and "Egg and egg products" only included one item each from the FFQ and were therefore called "Potatoes" and "Eggs". A new group was made for cod liver oil. The food groups included whole food items, not ingredients, as recipes were not used. The composition of the food groups is given in Additional file 1.

### Statistical analysis

Background characteristics of the study population are presented as mean and standard deviation (SD) or range for continuous variables, and proportion (%) for categorical variables. Single questions with predefined response options were treated as categorical variables, and calculated intake of food groups, energy, and nutrients as continuous variables. The reproducibility of single questions was evaluated by contingency tables for test-retest responses. The table diagonal represents the agreement, i.e. the responses in the same categories (test = retest). Total agreement (%) and agreement for the category "never/rarely" (%) were calculated for each table. Misclassification (%) was calculated for adjacent categories (± 1 and ± 2) and extreme opposite categories (lowest and highest). The symmetry of the misclassification was assessed by calculating the misclassification (%) on each side of the table diagonal (retest <test and retest> test). The difference across the diagonal indicates if there is a shift towards higher or lower responses in the retest compared to the test. The coefficients simple Kappa and weighted Kappa were also calculated and summarize the total agreement beyond that expected by chance [15].

For food groups, energy, and nutrients, we calculated the mean and standard deviation (SD) for the test and retest, the mean of the within person differences with both 95% confidence interval (± 2 SEM, i.e. standard error of the mean) and limits of agreement (± 2 SD). If the individual differences are normally distributed, 95% will lie within these limits [16]. We estimated Pearson's product moment correlation coefficient, *r*, and Spearman's rank correlation coefficient, *r*_*s*_. We also estimated the two intraclass correlation coefficients (ICCs) relevant to this reproducibility study with two measurements on every subject. Following the notation by Shrout and Fleiss [17],

 (a one-way random model)

 (a two-way mixed model).

The first number refers to one of three cases of random and fixed effects models used as examples in their paper. The second number indicates if the reliability is assessed for one single measurement, as in our case, or the mean of several measurements. The ICCs are based on variance decomposition, where BMS is the between-person mean square, WMS is the within-person mean square, and EMS is the residual mean square for the respective models. *ICC*(*1*, *1*) is a measure of the absolute agreement between the measurements, whereas *ICC*(*3*, *1*) should be interpreted in terms of consistency. This is because *ICC*(*3*, *1*) treats the variance between the two measurements as a fixed effect that does not contribute to the WMS.

To estimate the effects of measurement error in dietary intake on disease risk, we demonstrate the method of regression calibration using alcohol intake and reported high blood pressure in the questionnaire as an example. The idea behind regression calibration is to predict the true intake for each subject in the study, and to include the predicted value in a standard analysis to get corrected estimates. Alcohol was assumed to be measured with random, additive error, which was estimated from the test-retest replicates. Based on a linear calibration function for replicate data [18] the calibrated mean alcohol intake for each subject, , can be calculated as , where  is the grand mean of all bservations,  is the mean of the replicate measurements for each person, and *λ *is the reliability coefficient *ICC *(*1*, *2*) [17]. Alcohol (g/day) was then included as a continuous variable in a logistic regression model for high blood pressure (yes/no). Odds ratio (OR) estimates and 95% CIs were compared for the test, the retest, the test-retest mean, and the calibrated mean for 1 g and 10 g increases in alcohol intake. To avoid the influence of measurement errors in covariates we only present the crude estimates. Most analyses were done in SAS 8.2, but the ICCs with 95% CIs were calculated in SPSS 12.0. For the regression calibration we used the *rcal *program in STATA 8.0.

## Results

Table 1 shows that the selected characteristics of the respondents (n = 1488) and the total sample invited for the reproducibility study (n = 1994) were similar.

**Table 1:** Mean (range or SD^a^) or proportion for selected characteristics of the respondents and the total sample invited to the reproducibility study

**Characteristic**	**Respondents (n = 1488)^b^**		**Invited (n = 1994)^b^**	
Mean age, years (range)	59.9	(46–75)	59.8	(46–75)
Mean weight, kg (SD)	70.3	(12.0)	70.1	(12.0)
Mean height, cm (SD)	165.8	(5.8)	165.7	(5.7)
Mean BMI, kg/m^2 ^(SD)	25.6	(4.1)	25.5	(4.1)
Mean energy intake, MJ/day (SD)	6.44	(1.87)	6.48	(1.90)
Mean physical activity, scale 1–10 (SD) (1 = very low, 10 = very high)	5.3	(1.8)	5.2	(1.8)
Try to lose weight, %	36		35	
Daily smokers, %	23		24	
Teetotalers, %	12		13	
Take cod liver oil supplements, %	42		41	
College or university education, %	37		36	
Retired, %	25		24	

^a^Standard deviation

^b^Number may not total to 1488 or 1994 for each characteristic due to missing values

### Reproducibility of single food items

The food items in Table 2 were selected to illustrate the range of values for all the frequency questions in the FFQ. Reindeer meat and whole milk had the highest proportions of total agreement (≥85%), but also the highest agreement for the "never/rarely" consumption category (75–78%). Cod liver oil capsules, in winter and in the rest of the year, had the highest proportions of extreme misclassification, 5% and 12%, respectively. Oranges and the open categories "other vegetables", "other meat dishes" and "other fruit" had the lowest proportions of total agreement (34–40%), high proportions in the ± 2 adjacent categories (15–20%), and the lowest values for simple Kappa (0.20–0.25). Weighted Kappa was always higher, except for in reindeer meat where the weighed value was a little lower (0.57 compared to 0.58). The difference between the misclassification above and below the table diagonal was negative if more subjects reported a higher frequency in the retests compared to the test. The largest differences were observed for wine (-16%) and salad (-15%), followed by beer and meat chops (-9%). The difference was positive if more subjects reported a lower frequency in the retest compared to the test. The largest values were observed for oranges (38%), followed by carrots, swede, and chocolate (9–13%). Within the same food group (e.g. meat) the reports of some items increased (meat chops), while others decreased (roast). Similar for fish, salmon and shellfish increased while cod/coalfish decreased.

**Table 2:** Measures of reproducibility for single questions about consumption frequency in the test and retest food frequency questionnaire (FFQ)

		**Agreement, %**		**Misclassification, %**			**Symmetry of misclassification, %**			**Kappa**	
					
**Food item (response options)**	**n**	**Total**	**Never/rarely^a^**	**± 1 Categ.^b^**	**± 2 Categ.^b^**	**Extr.^c^**	**Retest<Test**	**Retest>Test**	**Difference**	**Simple**	**Weighted**
Potatoes (7)	1452	62	1	25	9	0.0	21	17	4	0.48	0.61
Carrots (7)	1406	43	1	42	11	0.0	35	22	13	0.30	0.54
Salad (7)	1169	41	3	41	12	0.0	22	37	-15	0.28	0.52
Swede (7)	1134	49	7	38	8	0.0	31	20	11	0.32	0.52
"Other vegetables" (7)	865	34	8	35	18	0.5	34	32	2	0.20	0.37
Oranges (7)	1107	39	5	34	20	0.1	50	12	38	0.25	0.47
"Other fruits" (7)	978	39	4	34	17	0.2	29	33	-4	0.25	0.42
Whole milk (6)	710	85	78	11	3	0.1	8	6	2	0.52	0.61
Meat, reindeer (6)	1339	86	75	11	1	0.6	8	6	2	0.58	0.57
Roast (beef, pork, lamb) (5)	1280	54	13	37	7	0.1	26	20	6	0.33	0.42
Meat, chops (5)	1301	62	10	35	4	0.0	15	24	-9	0.42	0.52
"Other meat dishes" (5)	921	40	11	41	15	0.2	31	28	3	0.21	0.32
Cod/coalfish/haddock/pollack (6)	1362	48	2	41	9	0.0	29	22	7	0.32	0.51
Salmon/trout (6)	1225	53	5	39	7	0.0	22	25	-3	0.35	0.48
Fish liver (5)	1229	78	43	18	3	0.2	12	10	2	0.68	0.77
Shellfish (4)	1374	70	35	27	2	0.1	13	17	-4	0.54	0.62
Beer (7)	1005	63	41	29	6	0.0	14	23	-9	0.46	0.61
Wine (7)	1137	46	13	40	13	0.0	19	35	-16	0.34	0.60
Chocolate (6)	1402	57	18	35	6	0.0	26	17	9	0.42	0.57
Cod liver oil capsules, winter (5)	156	76	3	13	3	5.1	9	15	-6	0.33	0.45
Cod liver oil capsules, rest of year (5)	130	69	15	12	4	11.5	15	16	-1	0.44	0.53

^a^Lowest response category in the FFQ, ^b^Categories: ± 1 or ± 2 from the line of agreement in the contingency table

^c^Extremes: test-retest responses in the lowest and highest categories

When the frequency questions were divided into categories, frequencies with no additional portion size question had a slightly higher median value for total agreement (65% versus 56%) and weighted Kappa (0.57 versus 0.51) than frequencies with a related portion size question. The median values for the portion size questions were 61% for total agreement and 0.47 for weighted Kappa (results not shown).

### Reproducibility of food groups

The mean daily intake of most food groups was slightly lower in the retest (Table 3). As seen from the 95% CI for the mean differences, significant decreases were observed for "potatoes", "fruits", "bread, crisp bread, breakfast cereal", "whole fish and shellfish", and "fat on bread". However, significant increases were observed for "red meat and chicken", "eggs", "orange juice, soft drinks, diluted syrups" and "alcoholic beverages". The limits of agreement were generally wide. Pearson's *r *ranged from 0.50 ("condiments and sauces for fish") to 0.79 ("cod liver oil supplements") with a median value of 0.66. *ICC*(*1*, *1*) and *ICC*(*3*, *1*) were very similar to *r*. The largest difference was seen for "alcoholic beverages" with *r *= 0.68, *ICC*(*1*, *1*) = 0.66, and *ICC*(*3*, *1*) = 0.67 (results not shown). Spearman's *r*_*s *_ranged from 0.55 ("bread, crisp bread and breakfast cereal") to 0.80 ("cream desserts and milk based puddings") with a median value of 0.70. There were some differences between *r*_*s *_and the other reliability coefficients, and for "dairy products" and "alcoholic beverages" *r*_*s *_was somewhat higher.

**Table 3:** Measures of reproducibility for the intake of food groups (g/day) in the test and retest food frequency questionnaire (FFQ), n = 1370

	**Test (FFQ_1_)**		**Retest (FFQ_2_)**		**Within person differences (FFQ_1_-FFQ_2_)**			**Pearson**		**Spearman**
					
**Food group (no. items)**	**Mean**	**SD^a^**	**Mean**	**SD**	**Mean**	**95% CI^b^**	**Mean ± 2 SD^c^**	***r***	**95% CI**	***r*_*s*_**
Potatoes (1)	107	56	103	57	3.4	(1.2, 5.7)	(-80, 87)	0.72	(0.69, 0.75)	0.70
Vegetables (7)	118	75	116	71	1.3	(-1.7, 4.4)	(-112, 115)	0.69	(0.66, 0.72)	0.71
Fruits (5)	200	127	185	130	15.4	(10.0, 20.8)	(-185, 216)	0.68	(0.65, 0.71)	0.71
Dairy products (12)	207	161	203	159	3.3	(-4.0, 10.6)	(-266, 273)	0.63	(0.60, 0.66)	0.76
Drinking milk, yogurt, cheese on bread (9)	181	158	179	154	2.4	(-4.8, 9.7)	(-264, 269)	0.62	(0.58, 0.65)	0.74
Cream desserts, milk based puddings (3)	25	25	25	26	0.8	(-0.2, 1.8)	(-36, 38)	0.72	(0.70, 0.75)	0.80
Cereal and cereal products (7)	161	65	154	63	7.0	(3.8, 10.2)	(-112, 126)	0.55	(0.52, 0.59)	0.55
Bread, crisp bread, breakfast cereal (5)	131	61	124	57	6.3	(3.3, 9.3)	(-103, 116)	0.55	(0.52, 0.59)	0.55
Pasta and rice (2)	30	23	30	22	0.7	(-0.2, 1.7)	(-36, 37)	0.66	(0.63, 0.69)	0.68
Meat and meat products (11)	96	46	95	45	0.2	(-2.0, 2.4)	(-81, 81)	0.59	(0.55, 0.62)	0.58
Read meat and chicken (5)	28	17	29	17	-1.1	(-1.9, -0.3)	(-31, 29)	0.62	(0.58, 0.65)	0.63
Processed meat (6)	68	37	67	37	1.3	(-0.6, 3.1)	(-66, 69)	0.57	(0.53, 0.60)	0.56
Fish and shellfish (17)	128	77	123	73	5.1	(1.9, 8.4)	(-116, 127)	0.66	(0.63, 0.69)	0.69
Whole fish (filets, steaks) and shellfish (8)	81	60	77	59	4.2	(1.4, 7.1)	(-100, 108)	0.60	(0.57, 0.63)	0.66
Fish products (9)	47	30	46	28	0.9	(-0.5, 2.3)	(-51, 53)	0.58	(0.54, 0.61)	0.59
Eggs (1)	14	11	14	11	-0.7	(-1.1, -0.2)	(-16, 15)	0.72	(0.69, 0.74)	0.70
Fat (margarine, butter) on bread (7)	12	13	11	11	0.8	(0.3, 1.2)	(-17, 18)	0.72	(0.70, 0.75)	0.78
Cakes (6)	35	26	36	27	-0.3	(-1.5, 0.9)	(-44, 43)	0.65	(0.62, 0.68)	0.65
Orange juice, soft drinks, diluted syrups (3)	106	121	112	130	-6.6	(-12.1, -1.0)	(-211, 198)	0.66	(0.62, 0.68)	0.70
Coffee (boiled, filtered, instant) (3)	368	249	366	258	1.5	(-9.3, 12.4)	(-400, 403)	0.67	(0.64, 0.70)	0.73
Alcoholic beverages (wine, beer, spirits) (3)	32	51	41	60	-9.1	(-11.5, -6.7)	(-98, 80)	0.68	(0.65, 0.71)	0.76
Condiments and sauces for fish (5)	9	9	9	9	0.3	(-0.2, 0.8)	(-18, 18)	0.50	(0.46, 0.54)	0.58
Sweets and salty snacks (6)	25	19	25	19	0.1	(-0.8, 0.9)	(-31, 31)	0.66	(0.63, 0.69)	0.71
Cod liver oil supplements (4)	2	3	2	3	-0.1	(-0.2, 0.0)	(-4, 4)	0.79	(0.77, 0.81)	0.74

^a^SD = Standard deviation, ^b^CI = Confidence interval, ^c^Referred to as "limits of agreement" in the text

### Reproducibility of energy and nutrients

The mean daily intake was significantly lower in the retest for energy, protein, fat (total and polyunsaturated), and total carbohydrate, but not the corresponding energy percentages (Table 4). Significant lower intakes were also observed for dietary fibre, retinol, vitamin C, and calcium. The intake was significantly higher in the retest for alcohol, and percent energy from both alcohol and sugar. Pearson's *r *ranged from 0.55 (calcium) to 0.78 (vitamin E), with a median value of 0.67. *ICC *(*1*, *1*), and *ICC *(*3*, *1*) were again very similar to *r *(results not shown). The range for Spearman's *r*_*s *_was 0.60 (protein, calcium) to 0.78 (% energy from alcohol) with a median of 0.67. *r*_*s *_was slightly higher than the other reliability coefficients for calcium, alcohol, and % energy from alcohol, which is consistent with the higher values observed for the food groups "dairy products" and "alcoholic beverages".

**Table 4:** Measures of reproducibility for the daily intake of energy and selected nutrients in the test and retest food frequency questionnaire (FFQ), n = 1370

	**Test (FFQ_1_)**		**Retest (FFQ_2_)**		**Within person differences (FFQ_1_-FFQ_2_)**			**Pearson**		**Spearman**
				
**Nutrient**	**Mean**	**SD^a^**	**Mean**	**SD**	**Mean**	**95% CI^b^**	**Mean ± 2 SD^c^**	***r***	**95% CI**	***r*_*s*_**
Energy (kJ)	6571	1748	6400	1732	170.2	(91.5, 249.0)	(-2801, 3142)	0.64	(0.60, 0.67)	0.63
Protein (g)	73.6	21.3	71.5	21.0	2.1	(1.0, 3.1)	(-36, 40)	0.60	(0.56, 0.63)	0.60
Total fat (g)	60.0	20.1	58.4	19.4	1.6	(0.7, 2.5)	(-32, 35)	0.63	(0.60, 0.66)	0.64
Polyunsaturated fat (g)	11.0	4.4	10.7	4.3	0.2	(0.1, 0.4)	(-7, 8)	0.65	(0.62, 0.68)	0.66
Total carbohydrate (g)	179.4	49.9	174.0	50.9	5.4	(3.2, 7.6)	(-77, 87)	0.67	(0.64, 0.70)	0.67
Dietary fiber (g)	19.6	6.2	18.6	5.9	1.0	(0.7, 1.2)	(-9, 11)	0.67	(0.64, 0.70)	0.66
Sugar (g)	22.1	13.4	22.3	13.7	-0.2	(-0.8, 0.4)	(-21, 21)	0.70	(0.67, 0.72)	0.71
Alcohol (g)	2.2	3.2	2.8	3.5	-0.6	(-0.7, -0.5)	(-6, 4)	0.72	(0.69, 0.75)	0.77
Retinol (RE^d^, μg)	1192	490	1142	482	50.3	(29.3, 71.3)	(-742, 843)	0.67	(0.64, 0.70)	0.66
Vitamin D (μg)	12.4	9.0	12.3	8.5	0.1	(-0.3, 0.5)	(-14, 14)	0.69	(0.66, 0.72)	0.70
Vitamin E (mg)	11.0	7.4	11.0	7.4	0.0	(-0.3, 0.3)	(-10, 10)	0.78	(0.76, 0.80)	0.73
Vitamin C (mg)	106	50	96	48	9.6	(7.5, 11.7)	(-69, 88)	0.68	(0.65, 0.70)	0.69
Calcium (mg)	624	246	600	248	23.2	(10.8, 35.6)	(-445, 492)	0.55	(0.51, 0.59)	0.60
% energy from protein	19.2	2.9	19.1	3.0	0.0	(-0.1, 0.2)	(-5, 5)	0.70	(0.67, 0.72)	0.69
% energy from fat	33.5	4.8	33.5	4.8	0.0	(-0.3, 0.2)	(-8, 8)	0.65	(0.62, 0.68)	0.65
% energy from carbohydrate	46.5	5.8	46.3	5.9	0.3	(0.0, 0.6)	(-10, 10)	0.64	(0.61, 0.67)	0.63
% energy from sugar	5.7	3.0	5.8	3.0	-0.2	(-0.3, -0.1)	(-5, 5)	0.68	(0.65, 0.71)	0.68
% energy from alcohol	1.0	1.6	1.4	1.8	-0.3	(-0.4, -0.2)	(-3, 2)	0.74	(0.72, 0.76)	0.78

^a^SD = Standard deviation, ^b^CI = Confidence interval, ^c^Referred to as limits of agreement in the text, ^d^Retinol equivalents

### Impact on diet-disease associations

In our example to demonstrate the regression calibration method, high blood pressure was negatively associated with alcohol intake (Table 5). The uncorrected estimates based on the test, the retest, and the test-retest mean were biased towards the null value (referred to as attenuation) having values closer to OR = 1 than the estimate corrected by regression calibration. The effect is more clearly seen for an increase of 10 g of alcohol per day (a little less than the amount in a standard glass of wine in NOWAC) with OR = 0.53 for the test, OR = 0.49 for the retest, OR = 0.45 for the test-retest mean, and OR = 0.38 for the calibrated intake.

**Table 5:** Odds ratio (OR) estimates and confidence intervals (CIs) for high blood pressure (yes = 301/no = 712) in relation to alcohol intake (g/day change) in the food requency uestionnaire (FFQ). Estimates are compared for the test, the retest, the test-retest mean, and calibrated mean intake, n = 1013

	**Test (FFQ_1_)**		**Retest (FFQ_2_)**		**Mean (FFQ_1 _and FFQ_2_)**		**Calibrated mean**	
			
**Alcohol intake (g/day)**	**OR**	**95% CI**	**OR**	**95% CI**	**OR**	**95% CI**	**OR**	**95% CI**
1	0.94	(0.90, 0.98)	0.93	(0.89, 0.97)	0.92	(0.88, 0.97)	0.91	(0.85, 0.97)
10	0.53	(0.33, 0.84)	0.49	(0.32, 0.75)	0.45	(0.28, 0.74)	0.38	(0.21, 0.67)

## Discussion

### Reproducibility of the FFQ

This study was designed to assess the test-retest reproducibility of the FFQ developed for the NOWAC study and the Norwegian part of the EPIC study. The response rate was relatively high (75%), and there were no indications of selection bias in the study sample. The estimated reliability coefficients for the intake of food groups and nutrients ranged from 0.5–0.8 with an approximate median value of 0.70.

Reproducibility studies of other self-administered FFQs designed to assess habitual diet over the past year, have reported median values between 0.6 and 0.7 for *r*_*s*_, *r*, or *ICC*(*1*, *1*) in Norwegian [19], Swedish [20,21], and Finnish women [22,23]. The reproducibility of the FFQs used by other EPIC centres is similar [24-27] or slightly higher with median values between 0.7 and 0.8 for *r*_*s *_or *r *[28,29].

In the studies cited above, the time period between administrations varied from 1–12 months. In our study the three month interval was expected to largely reflect variations associated with completing the questionnaire rather than changes in diet. However, recent food choices seem to have influenced the reporting of some foods, also referred to as seasonal reporting bias [30]. A strong indication of this is the high reported intake of oranges in the test FFQ, which was returned around Easter when oranges are traditionally eaten and marketed in Norway. The retest was returned in early summer, with much lower reports. Previous studies in Norway [19] and other countries [30] have also found the intake of citrus to be highly seasonal. Other differences that seem to reflect a change from a winter to a summer diet are the lower reports of typical winter vegetables (carrots and swede), and roast meat in the retest, and the higher reports of salad, wine, and meat chops, which are popular for outdoor barbequing. For oranges, the difference was sufficient to affect the mean intake of fruit and vitamin C. For other items, the differences seemed to cancel out within food groups (e.g. the vegetable and the meat groups). Although the results may have been influenced by the time of year the FFQ was administered, the significant differences observed were generally of a small magnitude.

The analysis of single food frequency questions confirms findings from other studies that recall is reliable for foods rarely eaten (whole milk and reindeer meat in our study population), and that misclassification is high for unspecific questions, such as "other vegetables", "other fruits" and "other meat dishes" [31]. The reproducibility of the food frequency questions also seemed to be influenced by the portion size questions. Lower median values for total agreement (%) and weighted Kappa were observed for frequencies when additional questions were asked about amounts. Another study has also found food frequency responses to be sensitive to whether only frequencies were filled in, or both frequencies and portion sizes [32]. However, changes in food frequency may be compensated by changes in portion size, and do not necessarily affect total food quantity.

### Statistical measures

Many FFQ reproducibility studies are undertaken as part of validation studies and tend to be analysed or presented in less detail. In the present study we evaluated the performance of each question. This is also helpful in the interpretation of food group and nutrient intake. When the responses are pre-coded, a categorical analysis is simple and does not require intake computations. Yet, we found few other examples in the literature [33]. In this study, weighted Kappa was generally higher than simple Kappa, indicating that most of the misclassification is found in the categories closest to the table diagonal.

For food groups, energy, and nutrients, *r *and *r*_*s *_are frequently reported reliability coefficients in the nutrition literature. They provide an assessment of the ranking of individuals, which is important for risk estimation in epidemiologic studies, but *r *is restricted to measuring linear associations and more sensitive to outliers than *r*_*s*_. This may give different values for *r *and *r*_*s*_, as observed in our study for the food groups "dairy products" and "alcoholic beverages". When outliers in the data were removed, *r *approached the value of *r*_*s*_. Log-transformation had the same effect (data not shown). If *r *and *r*_*s *_are similar, *r *is usually preferred, as it carries more information in terms of data variability. However, neither coefficient measures absolute agreement.

Intraclass correlation coefficients (ICCs) can be used as complementary or alternative measures to *r *or *r*_*s*_. The ICCs express proportions of variance and are therefore not restricted to linear associations or two replicates. Low *ICC*(*1*, *1*) can be interpreted as large within-person variation and low precision of measurements. However, *ICC*(*1*, *1*) also penalizes systematic error by giving a value that is lower than *r *[34]. We did not observe this in our data, but if *ICC*(*1*, *1*) and *r *were different, we would suggest presenting *ICC *(*1*, *1*) as a measure of absolute agreement, or both. In situations with more severe misclassification problems only one reliability coefficient may be insufficient, as different coefficients give different information [35]. *ICC*(*3*, *1*) does not penalize systematic errors and has been proposed in situation with systematic learning or fatigue effects, when this is not considered defects of the measurement instrument [36]. In our study we observed nearly identical values for *r*, *ICC*(*1*, *1*) and *ICC*(*3*, *1*). Thus, it would be interesting to examine how large the differences in mean and variance must be to generate larger discrepancies between the coefficients. But this was considered outside the scope of the paper.

### Measurement error effects

Given measures of reproducibility or validity, it can be difficult to predict how measurement errors in the data will affect disease risk estimates. Therefore, we calculated OR estimates for the association between high blood pressure and alcohol intake (g/day) based on the test and retest data (Table 5). This is a simple approach to investigate the impact of measurement errors, which has been used by others [37,38].

To correct the OR estimates for the within-person variability in the alcohol intake measurements, we used a linear calibration function for replicate data. Although alcohol had relatively high reproducibility (*r *= 0.72), the ORs based on the test, the retest, and the test-retest mean, were attenuated towards the null value compared to the ORs corrected by regression calibration. In general, the within-person variability or error is larger for single measurements than for the mean of replicate measurements, which in turn has larger variability than the calibrated mean. Large variability in the exposure data will often cause attenuation, or an underestimated association with the outcome. This is the most common effect of measurement errors, but the magnitude may be difficult to predict. There are also situations where bias can go in the opposite direction [18,39].

The association between alcohol intake and high blood pressure was here analyzed cross-sectionally in a logistic regression model without control of confounding factors. The example was primarily included to demonstrate the regression calibration method, which can be applied to any study design (cross-sectional-, case-control-, or cohort data), or regression model. But we think that the magnitude of the effect represents the weak diet-disease associations typically found in nutritional epidemiology. The calibration of dietary intake is usually based on validation studies to correct for systematic errors [20]. But as we demonstrate in the present study, reproducibility studies can also be used for calibration purposes to correct for random, additive error.

## Conclusion

In conclusion, the reproducibility of the dietary information from the FFQ used in the NOWAC study is within the range reported for similar instruments. However, the regression calibration showed that estimates of disease risk may be attenuated at this level of reproducibility. More knowledge of the type and magnitude of measurement errors and further development of correction methods could give us more accurate dietary intake levels and disease risk estimates in the future.

## Competing interests

The author(s) declare that they have no competing interests.

## Authors' contributions

CLP cleaned the data, calculated the dietary intake, performed the statistical analysis, and drafted the manuscript. EL is the principal investigator in the Norwegian Women and Cancer Study and conceived and designed the present study in collaboration with AH. MBV and PL contributed to the statistical analysis. The manuscript was revised by AH and MBV. All authors read and approved the final version.

## Supplementary Material

Additional File 1 Additional file 1 Food items included in the dietary intake computations listed by the food groups in Table 3 Click here for file

Additional File 2 Additional file 2 Original version of the food frequency questionnaire used in the reproducibility study Click here for file
