# Supplementary material for: Test-retest reproducibility of a food frequency questionnaire (FFQ) and estimated effects on disease risk in the Norwegian Women and Cancer Study (NOWAC)
Source: Nutr J. 2006 Jan 31;5:4. doi: 10.1186/1475-2891-5-4 (PMC1434764; doi:10.1186/1475-2891-5-4)
Supplement: Additional File 1 — Food items included in the dietary intake computations listed by the food groups in Table 3 [file 1475-2891-5-4-S1.pdf]

Appendix 1 - Food items included in the calculation of food and nutrient intake listed by the food groups in Table 3

Food group (number of items)[#]	Description of items in group[*]
**Potatoes (1)**	potatoes (boiled, fried, mashed)
**Vegetables (7)**	carrots[*], cabbage[*], swede[*], cauliflower/broccoli[*], mixed salad[*], frozen vegetable mix[*], "other vegetables"
**Fruits (5)**	apples/pears, oranges, bananas, "other fruits", compote/canned fruit
**Dairy products (12)**	
Drinking milk, yogurt, cheese on bread (9)	whole milk (fresh, sour), low fat milk (fresh, sour), extra low fat milk, skimmed milk (fresh, sour), yoghurt, white cheese (full fat), white cheese (low fat), whey cheese (full fat), whey cheese (low fat)
Cream desserts, milk based puddings (3)	chocolate pudding/crème caramel, cream rice/mousse, rice porridge
**Cereal and cereal products (7)**	
Bread, crisp bread, breakfast cereal (5)	bread (coarse), bread (semi coarse), white bread, crispbread, cereal/oatmeal/muesli
Pasta and rice (2)	rice, spaghetti/macaroni
**Meat and meat products (11)**	
Read meat and chicken (5)	roast (beef, pork, lamb)[*], chops[*], steak, chicken, reindeer meat
Processed meat (6)	meatballs/hamburger[*], hot dogs[*], casseroles[*], pizza with meat[*], "other meat dishes", sandwich meats/liver paste
**Fish and shellfish (17)**	
Whole fish (filets, steaks) and shellfish (8)	poached cod/coalfish/haddock/pollack[*], fried cod/coalfish/haddock/pollack[*], catfish/flounder/redfish[*], salmon/trout[*], mackerel[*], herring[*], "other fish"[*], shellfish
Fish products (9)	fishcake/pudding/balls[*], fish gratin[*], fish sticks[*], "other fish dishes", cod roe, cod liver[*], mackerel in tomato sauce/smoked mackerel, caviar, "other fish spreads"
**Eggs (1)**	Eggs (fried, boiled, scrambled, omelet)

Food group (items)	Description of items in group
**Fat on bread (7)[#]**	butter*, hard margarine (e.g. Per, Melange)*, soft margarine (e.g. Soft, Vita, Solsikke)*, margarine mixed with butter (e.g. Bremyk)*, “Brelett”*, light margarine (e.g. Soft light, Letta)*, medium light margarine (e.g. Olivero, Omega)*
**Cakes (6)**	buns, Danish pastries, cakes, pancakes, waffles, sweet biscuits
**Orange juice, soft drinks, diluted syrups (3)**	orange juice, soft drinks/syrups with sugar, soft drinks/syrups without sugar
**Coffee (3)**	boiled, filtered, instant
**Alcoholic beverages (3)**	beer, wine, spirits
**Condiments and sauces for fish (5)**	butter/margarine*, sour cream (35% fat)*, sour cream (20% fat)*, sauce (white/brown) with fat*, sauce (white/brown) without fat*
**Sweets and salty snacks (6)**	jam/sweet sandwich spreads, ice cream in summer*, ice cream in rest of year*, chocolate*, potato chips, peanuts
**Cod liver oil supplements (4)**	cod liver oil (liquid) in winter*, and in rest of year* cod liver oil (capsules) in winter*, and in rest of year*

[#]All items in the table correspond to a frequency question in the FFQ, except for fat on bread, which was a question about types of fat (one or more) used. For those who reported to use fat on bread, the consumption frequency was the same as for bread.

*Items with a separate portion size question in the FFQ to determine the usual amount consumed.
The amounts of the 42 items marked in the table were estimated from 28 portion size questions, since one portion size was sometimes used for multiple items:

- For whole fish, two portion sizes (for poached and fried fish) were used for seven types of fish.
- For fat on bread, one portion size was included, but seven types could be specified.
- For season specific frequencies, the same portion size was used for ice cream in summer, and ice cream in rest of year. This was also the case for cod liver oil supplements where the amounts of liquid oil and capsules were used for both in winter and in rest of year.
