# Supplementary material for: Test-retest reproducibility of a food frequency questionnaire (FFQ) and estimated effects on disease risk in the Norwegian Women and Cancer Study (NOWAC)
Source: Nutr J. 2006 Jan 31;5:4. doi: 10.1186/1475-2891-5-4 (PMC1434764; doi:10.1186/1475-2891-5-4)
Supplement: Additional File 2 — Original version of the food frequency questionnaire used in the reproducibility study [file 1475-2891-5-4-S2.pdf]

Vi ber deg angi din fysiske aktivitet etter en skala fra svært lite til svært mye. Skalaen nedenfor går fra 1-10. Med fysisk aktivitet mener vi både arbeid i hjemmet og i yrkeslivet, samt trening og annen fysisk aktivitet som turgåing o.l. Sett kryss over det tallet som best angir ditt nivå av fysisk aktivitet.

Alder	Svært lite									Svært mye
30 år	1	2	3	4	5	6	7	8	9	10
I dag	1	2	3	4	5	6	7	8	9	10

Høyde og vekt

Hvor høy er du? (i hele cm.) ☐

Hvor mye veier du i dag? (i hele kg.) ☐

Kosthold

Påvirker noen av følgende forhold kostholdet ditt? (sett gjerne flere kryss)

- ☐ Er vegetarianer/veganer
- ☐ Har anoreksi
- ☐ Spiser ikke norsk kost til daglig
- ☐ Har allergi/intoleranse
- ☐ Har bulimi
- ☐ Kronisk sykdom
- ☐ Prøver å gå ned i vekt

Vi er interessert i å få kjennskap til hvordan kostholdet ditt er vanligvis. Kryss av for hvert spørsmål om hvor ofte du i gjennomsnitt siste året har brukt den aktuelle matvaren, og hvor mye du pleier å spise/drikke hver gang.

Hvor mange glass melk drikker du vanligvis av hver type? (Sett ett kryss pr. linje)

	aldri/ sjelden	1-4 pr. uke	5-6 pr. uke	1 pr. dag	2-3 pr. dag	4+ pr. dag
Helmelk (søt, sur)	☐	☐	☐	☐	☐	☐
Lettmelk (søt, sur)	☐	☐	☐	☐	☐	☐
Ekstra lettmelk	☐	☐	☐	☐	☐	☐
Skummet (søt, sur)	☐	☐	☐	☐	☐	☐

Hvor mange kopper kaffe drikker du vanligvis av hver sort? (Sett ett kryss for hver linje)

	aldri/ sjelden	1-6 pr. uke	1 pr. dag	2-3 pr. dag	4-5 pr. dag	6-7 pr. dag	8+ pr. dag
Kokekaffe	☐	☐	☐	☐	☐	☐	☐
Traktekaffe	☐	☐	☐	☐	☐	☐	☐
Pulverkaffe	☐	☐	☐	☐	☐	☐	☐

Hvor mange glass appelsinjuice, saft og brus drikker du vanligvis? (Sett ett kryss for hver linje)

	aldri/ sjelden	1-3 pr. uke	4-6 pr. uke	1 pr. dag	2-3 pr. dag	4+ pr. dag
Appelsinjuice	☐	☐	☐	☐	☐	☐
Saft/brus med sukker	☐	☐	☐	☐	☐	☐
Saft/brus sukkerfri	☐	☐	☐	☐	☐	☐

Hvor ofte spiser du yoghurt (1 beger)? (Sett ett kryss)

- ☐ Aldri/sjelden
- ☐ 2-3 pr. uke
- ☐ 1 pr. uke
- ☐ 4+ pr. uke

Hvor ofte har du i gjennomsnitt siste året spist kornblanding, havregryn eller müsli? (Sett ett kryss)

- ☐ Aldri/sjelden
- ☐ 4-6 pr. uke
- ☐ 1-3 pr. uke
- ☐ 1 pr. dag

Hvor mange skiver brød/rundstykker og knekkebrød/skonrokker spiser du vanligvis? (1/2 rundstykke = 1 brødskive) (Sett ett kryss for hver linje)

	aldri/ sjelden	1-4 pr. uke	5-7 pr. uke	2-3 pr. dag	4-5 pr. dag	6+ pr. dag
Grovt brød	☐	☐	☐	☐	☐	☐
Kneipp/halvfint	☐	☐	☐	☐	☐	☐
Fint brød	☐	☐	☐	☐	☐	☐
Knekkebrød o.l.	☐	☐	☐	☐	☐	☐

Nedenfor er det spørsmål om bruk av ulike påleggstyper. Vi spør om hvor mange brødskiver med det aktuelle pålegget du pleier å spise. Dersom du også bruker matvarene i andre sammenhenger enn til brød (f. eks. til vafler, frokostblandinger, grøt), ber vi om at du tar med dette når du besvarer spørsmålene.

På hvor mange brødskiver bruker du? (Sett ett kryss pr. linje)

	0 pr. uke	1-3 pr. uke	4-6 pr. uke	1 pr. dag	2-3 pr. dag	4+ pr. dag
Syltetøy og annet søtt pålegg	☐	☐	☐	☐	☐	☐
Brun ost, helfet	☐	☐	☐	☐	☐	☐
Brunost, halvfet/mager	☐	☐	☐	☐	☐	☐
Hvitost, helfet	☐	☐	☐	☐	☐	☐
Hvitost, halvfet/mager	☐	☐	☐	☐	☐	☐
Kjøttpålegg, Leverpostei	☐	☐	☐	☐	☐	☐

På hvor mange brødskiver pr. uke har du i gjennomsnitt siste året spist? (Sett ett kryss pr. linje)

	0 pr. uke	1 pr.uke	2-3 pr.uke	4-6 pr.uke	7-9 pr.uke	10+ pr.uke
Makrell i tomat, røkt makrell	☐	☐	☐	☐	☐	☐
Kaviar	☐	☐	☐	☐	☐	☐
Annet fiskepålegg	☐	☐	☐	☐	☐	☐

Hva slags fett bruker du vanligvis på brødet? (Sett gjerne flere kryss)

- ☐ Bruker ikke fett på brødet
- ☐ Smør
- ☐ Hard margarin (f. eks. Per, Melange)
- ☐ Myk margarin (f. eks. Soft, Vita og Solsikke)
- ☐ Smørblandet margarin (f. eks. Bremyk)
- ☐ Brelett
- ☐ Lettmargarin (f. eks. Soft light, Letta)
- ☐ Middels lett margarin (f. eks. Olivero, Omega)

Dersom du bruker fett på brødet, hvor tykt lag pleier du smøre på? (En kuvertpakke med margarin veier 12 gram). (Sett ett kryss)

- ☐ Skrapet (3 g)
- ☐ Godt dekket (8 g)
- ☐ Tynt lag (5 g)
- ☐ Tykt lag (12 g)

Hvor ofte spiser du frukt? (Sett ett kryss pr. linje)

	aldri/ sjelden	1-3 pr.mnd.	1 pr.uke	2-4 pr.uke	5-6 pr.uke	1 pr.dag	2+ pr. dag
Epler/pærer	☐	☐	☐	☐	☐	☐	☐
Appelsiner o.l.	☐	☐	☐	☐	☐	☐	☐
Bananer	☐	☐	☐	☐	☐	☐	☐
Annen frukt	☐	☐	☐	☐	☐	☐	☐

Hvor ofte spiser du ulike typer grønnsaker?
(Sett ett kryss pr. linje)

	aldri/ sjelden	1-3 pr.mnd.	1 pr.uke	2 pr.uke	3 pr.uke	4-5 pr.uke	6-7 pr. uke
Gulrøtter	☐	☐	☐	☐	☐	☐	☐
Kål	☐	☐	☐	☐	☐	☐	☐
Kålrot	☐	☐	☐	☐	☐	☐	☐
Brokkoli/blomkål	☐	☐	☐	☐	☐	☐	☐
Blandet salat	☐	☐	☐	☐	☐	☐	☐
Grønnsakblanding (frossen)	☐	☐	☐	☐	☐	☐	☐
Andre grønnsaker	☐	☐	☐	☐	☐	☐	☐

For de grønnsakene du spiser, kryss av for hvor mye du spiser hver gang. (Sett ett kryss for hver sort)

- gulrøtter	☐ 1/2 stk.	☐ 1 stk.	☐ 1 1/2 stk.	☐ 2+ stk.
- kål	☐ 1/2 dl	☐ 1 dl	☐ 1 1/2 dl	☐ 2+ dl
- kålrot	☐ 1/2 dl	☐ 1 dl	☐ 1 1/2 dl	☐ 2+ dl
- brokkoli/blomkål	☐ 1-2 buketter	☐ 3-4 buketter		☐ 5+ buketter
- blandet salat	☐ 1 dl	☐ 2 dl	☐ 3 dl	☐ 4+ dl
- grønnsakblanding	☐ 1/2 dl	☐ 1 dl	☐ 2 dl	☐ 3+ dl

Hvor mange poteter spiser du vanligvis (kokte, stekte, mos)? (Sett ett kryss)

☐ Spiser ikke/spiser sjelden poteter
☐ 1-4 pr. uke ☐ 5-6 pr. uke ☐ 1 pr. dag ☐ 2 pr. dag
☐ 3 pr. dag ☐ 4+ pr. dag

Hvor ofte bruker du ris og spagetti/makaroni ?
(Sett ett kryss pr. linje)

	aldri/ sjelden	1-3 pr. mnd.	1 pr. uke	2 pr. uke	3+ pr. uke
Ris	☐	☐	☐	☐	☐
Spagetti, makaroni	☐	☐	☐	☐	☐

Hvor ofte spiser du risengrynsgrøt? (Sett ett kryss)

Aldri/sjelden	☐	1 pr. mnd.	☐
2-3 pr. mnd.	☐	1+ pr. uke	☐

Hva slags fett blir vanligvis brukt til matlaging i din husholdning? (Sett gjerne flere kryss)

Smør	☐	Myk margarin (eks.Soft)	☐
Hard margarin (eks. Melange)	☐	Smørblandet margarin (eks. Bremyk)	☐
Flytende margarin	☐	Soyaolje	☐
Olivenolje	☐	Maisolje	☐

Fisk

Vi vil gjerne vite hvor ofte du pleier å spise fisk, og ber deg fylle ut spørsmålene om fiskeforbruk så godt du kan. Tilgangen på fisk kan variere gjennom året. Vær vennlig å markere i hvilke årstider du spiser de ulike fiskeslagene.

	aldri/ sjelden	like mye hele året	vintrer	vår	sommer	høst
Torsk, sei, hyse, lyr	☐	☐	☐	☐	☐	☐
Steinbit, flyndre, uer	☐	☐	☐	☐	☐	☐
Laks, ørret	☐	☐	☐	☐	☐	☐
Makrell	☐	☐	☐	☐	☐	☐
Sild	☐	☐	☐	☐	☐	☐
Annen fisk	☐	☐	☐	☐	☐	☐

Med tanke på de periodene av året der du spiser fisk, hvor ofte pleier du å spise følgende?
(Sett ett kryss pr. linje)

	aldri/ sjelden	1 pr. mnd.	2-3 pr. mnd.	1 pr. uke	2 pr. uke	3+ pr. uke
Kokt torsk, sei, hyse, lyr	☐	☐	☐	☐	☐	☐
Stekt torsk, sei, hyse, lyr	☐	☐	☐	☐	☐	☐
Steinbit, flyndre, uer	☐	☐	☐	☐	☐	☐
Laks, ørret	☐	☐	☐	☐	☐	☐
Makrell	☐	☐	☐	☐	☐	☐
Sild	☐	☐	☐	☐	☐	☐
Annen fisk	☐	☐	☐	☐	☐	☐

Dersom du spiser fisk, hvor mye spiser du vanligvis pr. gang? (1 skive/stykke = 150 gram)

Kokt fisk (skive)
☐ 1 ☐ 1,5 ☐ 2 ☐ 3+

Stekt fisk (stykke)
☐ 1 ☐ 1,5 ☐ 2 ☐ 3+

Hvor mange ganger pr. år spiser du fiskeinnmat?
(Sett ett kryss pr. linje)

	0	1-3	4-6	7-9	10+
Rogn	☐	☐	☐	☐	☐
Fiskelever	☐	☐	☐	☐	☐

Dersom du spiser fiskelever, hvor mange spiseskjeer pleier du å spise hver gang? (Sett ett kryss)

1	2	3-4	5-6	7+
☐	☐	☐	☐	☐

Hvor ofte bruker du følgende typer fiskemat?
(Sett ett kryss pr. linje)

	aldri/ sjelden	1 pr. mnd.	2-3 pr. mnd.	1 pr. uke	2+ pr. uke
Fiskekaker/pudding/boller	☐	☐	☐	☐	☐
Plukkfisk/fiskegrateng	☐	☐	☐	☐	☐
Frityrfisk/fiskepinner	☐	☐	☐	☐	☐
Andre fiskeretter	☐	☐	☐	☐	☐

Hvor stor mengde pleier du vanligvis å spise av de ulike rettene? (Sett ett kryss for hver linje)

- fiskekaker/pudding/boller (stk.) (2 fiskeboller=1 fiskekake)	☐ 1	☐ 2	☐ 3	☐ 4+
- plukkfisk, fiskegrateng (dl)	☐ 1-2	☐ 3-4	☐ 5+	
- frityrfisk, fiskepinner (stk.)	☐ 1-2	☐ 3-4	☐ 5-6	☐ 7+

Hvor ofte spiser du skalldyr (f. eks. reker, krabbe)**?**
(Sett ett kryss)

☐ Aldri/sjelden ☐ 1 pr. mnd ☐ 2-3 pr. mnd ☐ 1+ pr. uke

I tillegg til informasjon om fiskeforbruk er det viktig å få kartlagt hvilket tilbehør som blir servert til fisk. Hvor ofte bruker du følgende til fisk? (Sett ett kryss pr. linje)

	aldri/ sjelden	1 pr. mnd.	2-3 pr. mnd.	1 pr. uke	2+ pr. uke
Smeltet eller fast margarin/fett	☐	☐	☐	☐	☐
Seterrømme (35%)	☐	☐	☐	☐	☐
Lettrømme (20%)	☐	☐	☐	☐	☐
Saus med fett (hvit/brun)	☐	☐	☐	☐	☐
Saus uten fett (hvit/brun)	☐	☐	☐	☐	☐

For de ulike typene tilbehør du bruker til fisk, vær vennlig å kryss av for hvor mye du vanligvis pleier spise.

- smeltet/fast fett (ss)	☐ 1/2	☐ 1	☐ 2	☐ 3	☐ 4+
- seterrømme (ss)	☐ 1/2	☐ 1	☐ 2	☐ 3	☐ 4+
- lettrømme (ss)	☐ 1/2	☐ 1	☐ 2	☐ 3	☐ 4+
- saus med fett (dl)	☐ 1/4	☐ 1/2	☐ 3/4	☐ 1	☐ 2+
- saus uten fett (dl)	☐ 1/4	☐ 1/2	☐ 3/4	☐ 1	☐ 2+

Andre matvarer

Hvor ofte spiser du reinkjøtt?

☐ Aldri/sjelden ☐ 1 pr. mnd. ☐ 2-3 pr. mnd. ☐ 1 pr. uke
☐ 2-3 pr. uke ☐ 4+ pr. uke

Hvor ofte spiser du følgende kjøtt- og fjærkreretter?
(Sett ett kryss for hver rett)

	aldri/ sjelden	1 pr.mnd.	2-3 pr.mnd.	1 pr.uke	2+ pr.uke
Steik (okse, svin, får)	☐	☐	☐	☐	☐
Koteletter	☐	☐	☐	☐	☐
Biff	☐	☐	☐	☐	☐
Kjøttkaker, karbonader	☐	☐	☐	☐	☐
Pølser	☐	☐	☐	☐	☐
Gryterett, lapskaus	☐	☐	☐	☐	☐
Pizza med kjøtt	☐	☐	☐	☐	☐
Kylling	☐	☐	☐	☐	☐
Andre kjøttretter	☐	☐	☐	☐	☐

Dersom du spiser følgende retter, oppgi mengden du vanligvis spiser: (Sett ett kryss for hver linje)

- steik (skiver)	☐ 1	☐ 2	☐ 3	☐ 4+
- koteletter (stk.)	☐ 1/2	☐ 1	☐ 1,5	☐ 2+
- kjøttkaker, karbonader (stk.)	☐ 1	☐ 2	☐ 3	☐ 4+
- pølser (stk. à 150g)	☐ 1/2	☐ 1	☐ 1,5	☐ 2+
- gryterett, lapskaus (dl)	☐ 1-2	☐ 3	☐ 4	☐ 5+
- pizza m/kjøtt (stykke à 100 g)	☐ 1	☐ 2	☐ 3	☐ 4+

Hvor mange egg spiser du vanligvis i løpet av en uke?(stekte, kokte, eggerøre, omelett) (Sett ett kryss)

☐ 0 ☐ 1 ☐ 2 ☐ 3-4
☐ 5-6 ☐ 7+

Vi ber deg fylle ut hovedrettene til middag en gang til som en oppsummering. Kryss av i den ruten som passer hvor ofte du i gjennomsnitt i løpet av siste år har spist slik mat til middag

	5+ pr.uke	4 pr.uke	3 pr.uke	2 pr.uke	1 pr.uke	2-3 pr.mnd.	1 pr.mnd.	nesten aldri
Rent kjøtt	☐	☐	☐	☐	☐	☐	☐	☐
Oppmalt kjøtt	☐	☐	☐	☐	☐	☐	☐	☐
Fet fisk (makrell, laks o.l.)	☐	☐	☐	☐	☐	☐	☐	☐
Mager fisk (torsk o.l.)	☐	☐	☐	☐	☐	☐	☐	☐
Fiskemat	☐	☐	☐	☐	☐	☐	☐	☐

Hvor ofte spiser du iskrem? (til dessert, krone-is osv.)
Sett et kryss for hvor ofte du spiser iskrem om sommeren, og et kryss for resten av året)

	aldri/ sjelden	1 pr. mnd.	2-3 pr. mnd.	1 pr. uke	2+ pr. uke
-Om sommeren	☐	☐	☐	☐	☐
-Resten av året	☐	☐	☐	☐	☐

Hvor mye is spiser du vanligvis pr. gang? (Sett ett kryss)

☐ 1dl ☐ 2 dl ☐ 3 dl ☐ 4+ dl

Hvor ofte spiser du bakevarer som boller kaker, wienerbrød eller småkaker (Sett ett kryss pr. linje)

	aldri/ sjelden	1-3 mnd.	1 pr. uke	2-3 pr uke	4-6 pr. uke	7+ pr. uke
Gjærbakst (boller)	☐	☐	☐	☐	☐	☐
Wienerbrød	☐	☐	☐	☐	☐	☐
Kaker	☐	☐	☐	☐	☐	☐
Pannekaker	☐	☐	☐	☐	☐	☐
Vafler	☐	☐	☐	☐	☐	☐
Småkaker	☐	☐	☐	☐	☐	☐

Hvor ofte spiser du dessert? (Sett ett kryss pr. linje)

	aldri/ sjelden	1-3 mnd.	1 pr. uke	2-3 pr uke	4-6 pr. uke	7+ pr. uke
Pudding sjokolade/karamell	☐	☐	☐	☐	☐	☐
Riskrem, fromasj	☐	☐	☐	☐	☐	☐
Kompott, fruktgrøt, hermetisk frukt	☐	☐	☐	☐	☐	☐

Hvor ofte spiser du sjokolade? (Sett ett kryss)

☐ aldri/sjelden ☐ 1-3 pr. mnd ☐ 1 pr. uke
☐ 2-3 pr. uke ☐ 4-6 pr. uke ☐ 1+ pr. dag

Dersom du spiser sjokolade, hvor mye pleier du vanligvis å spise hver gang? Tenk deg størrelsen på en Kvikk-Lunsj sjokolade, og oppgi hvor mye du spiser i forhold til den.

☐ 1/4 ☐ 1/2 ☐ 3/4 ☐ 1 ☐ 1,5 ☐ 2+

Hvor ofte spiser du salt snacks? (Sett ett kryss)

	aldri/ sjelden	1-3 pr. mnd.	1 pr. uke	2-3 pr. uke	4-6 pr. uke	7+ pr. uke
Potetchips	☐	☐	☐	☐	☐	☐
Peanøtter	☐	☐	☐	☐	☐	☐

Tilberedningsmåte

Har du mikrobølgeovn? Ja ☐ Nei ☐

Hvis Ja; hvor mange ganger pr. uke bruker du mikrobølgeovnen til

middagslaging (ant. ganger) ☐

annet (ant. ganger) ☐

Hvilken farge foretrekker du på stekeskorpen?

☐ Lys brun ☐ Middels brun ☐ Mørk brun

Hvor ofte spiser du stekt eller grillet mat?

	aldri/ sjelden	1-3 pr. mnd.	1 pr. uke	2-3 pr. uke	4-6 pr. uke	7+ pr. uke
Mørkt kjøtt (biff o.l.)	☐	☐	☐	☐	☐	☐
Lyst kjøtt (kylling)	☐	☐	☐	☐	☐	☐
Oppmalt kjøtt (kjøttkaker o.l.)	☐	☐	☐	☐	☐	☐
Bacon	☐	☐	☐	☐	☐	☐
Fisk	☐	☐	☐	☐	☐	☐

Bruker du stekefettet eller sjyen etter steking?

☐ nei, aldri ☐ av og til ☐ som oftest ☐ ja, alltid

Tran og fiskeoljekapsler

Bruker du tran (flytende)? Ja ☐ Nei ☐

Hvis ja; hvor ofte tar du tran?
Sett ett kryss for hver linje.

	aldri/ sjelden	1-3 pr. mnd.	1 pr. uke	2-6 pr. uke	daglig
Om vinteren	☐	☐	☐	☐	☐
Resten av året	☐	☐	☐	☐	☐

Hvor mye tran pleier du å ta hver gang?

☐ 1 ts. ☐ 1/2 ss. ☐ 1+ ss.

Bruker du tranpiller/kapsler? Ja ☐ Nei ☐

Hvis ja; hvor ofte tar du tranpiller/kapsler?
Sett ett kryss for hver linje.

	aldri/ sjelden	1-3 pr. mnd.	1 pr. uke	2-6 pr. uke	daglig
Om vinteren	☐	☐	☐	☐	☐
Resten av året	☐	☐	☐	☐	☐

Hvilken type tranpiller/kapsler bruker du vanligvis, og hvor mange pleier du å ta hver gang?

Navn ☐ ant. ☐

Bruker du fiskeoljekapsler? (omega-3) Ja ☐ Nei ☐

Hvis ja; hvor ofte tar du fiskeoljekapsler?

aldri/ sjelden	1-3 pr. mnd.	1 pr. uke	2-6 pr. uke	daglig
☐	☐	☐	☐	☐

Hvilken type fiskeoljekapsler bruker du vanligvis, og hvor mange pleier du å ta hver gang?

Navn ☐ ant. ☐

Kosttilskudd og alternativ medisin

Hvor ofte bruker du følgende typer tilskudd til kostholdet? (Sett ett kryss pr. linje)

Navn på vitamin/mineraltilskudd:	aldri/ sjelden	1-3 pr. mnd.	1 pr. uke	2-6 pr. uke	daglig
	☐	☐	☐	☐	☐
	☐	☐	☐	☐	☐
	☐	☐	☐	☐	☐
	☐	☐	☐	☐	☐

Bruker du naturpreparater? Ja ☐ Nei ☐

Hvis ja; Når brukte du det sist? År ☐

Bruker du soyapreparater? Ja ☐ Nei ☐

Hvis ja; Når brukte du det sist? År ☐

Har du brukt homeopatiske midler? Ja ☐ Nei ☐

Hvis ja; Når brukte du det sist? År ☐

Alkohol

Er du totalavholdskvinne? Ja ☐ Nei ☐

Hvis Nei, hvor ofte og hvor mye drakk du i gjennomsnitt siste året? (Sett ett kryss for hver linje)

	aldri/ sjelden	1 pr. mnd.	2-3 pr. mnd.	1 pr. uke	2-4 pr. uke	5-6 pr. uke	1+ pr. dag
Øl (1/2 l.)	☐	☐	☐	☐	☐	☐	☐
Vin (glass)	☐	☐	☐	☐	☐	☐	☐
Brennevin (drink)	☐	☐	☐	☐	☐	☐	☐
